# Resveratrol ameliorates myocardial fibrosis by inhibiting ROS/ERK/TGF-β/periostin pathway in STZ-induced diabetic mice

**DOI:** 10.1186/s12872-015-0169-z

**Published:** 2016-01-11

**Authors:** Han Wu, Guan-Nan Li, Jun Xie, Ran Li, Qin-Hua Chen, Jian-Zhou Chen, Zhong-Hai Wei, Li-Na Kang, Biao Xu

**Affiliations:** Department of Cardiology, Drum Tower Hospital, Nanjing University Medical School, Nanjing, 210008 China

**Keywords:** Diabetes, Resveratrol, Periostin, Fibrosis, Oxidative stress

## Abstract

**Background:**

Myocardial fibrosis is an essential hallmark of diabetic cardiomyopathy (DCM) contributing to cardiac dysfunctions. Resveratrol, an antioxidant, exerts its anti-fibrotic effect via inhibition of oxidative stress, while the underlying molecular mechanism remains largely elusive. Periostin, a fibrogenesis matricellular protein, has been shown to be associated with oxidative stress. In the present study, we investigated the role of periostin in anti-fibrotic effect of resveratrol in streptozocin (STZ)-induced diabetic heart and the underlying mechanisms.

**Methods:**

Diabetic mice were induced by STZ injection. After treatment with resveratrol (5 or 25 mg/kg/day i.g) or Saline containing 0.5 % carboxymethyl cellulose (CMC) for 2 months, the hearts were detected for oxidative stress and cardiac fibrosis using western blot, Masson’s trichrome staining and Dihydroethidium (DHE) staining. In in vitro experiments, proliferation and differentiation of fibroblasts under different conditions were investigated through western blot, 3-(4,5)-dimethylthiahiazo (−z-y1)-3,5-di-phenytetrazoliumromide (MTT) assay and immunofluorescence staining.

**Results:**

Administration of resveratrol significantly mitigated oxidative level, interstitial fibrosis and expressions of related proteins in STZ-induced diabetic hearts. In in vitro experiments, resveratrol exhibited anti-proliferative effect on primary mouse cardiac fibroblasts via inhibiting reactive oxygen species (ROS)/extracellular regulated kinase (ERK) pathway and ameliorated myofibroblast differentiation via suppressing ROS/ERK/ transforming growth factor β (TGF-β)/periostin pathway.

**Conclusion:**

Increased ROS production, activation of ERK/TGF-β/periostin pathway and myocardial fibrosis are important events in DCM. Alleviated ROS genesis by resveratrol prevents myocardial fibrosis by regulating periostin related signaling pathway. Thus, inhibition of ROS/periostin may represent a novel approach for resveratrol to reverse fibrosis in DCM.

## Background

Diabetes mellitus (DM) is a common metabolic disease, and cardiovascular diseases have become the principal culprits leading to mortality in diabetic patients. An accumulating body of evidence suggests that DCM characterized by both early-onset diastolic and late-onset systolic dysfunctions independent of hypertension and coronary artery disease is one of the major causes leading to heart failure in diabetic patients. Despite extensive studies, a thorough understanding of the pathogenesis has remained elusive and there are no effective therapies for them.

Mechanistically, complex and highly diversified mechanisms are involved in the pathogenesis of DCM, but of particular importance is myocardial fibrosis. Interstitial fibrosis of myocardium is characterized by increased matrix proteins accumulation due to an effect of profibrotic growth factor, especially TGF-β. Periostin, a matricellular protein, plays a critical role in regulating fibrogenesis of various diseases, such as heart failure [1, 2], myocardial infarction [3] and idiopathic pulmonary fibrosis [4]. Periostin can be stimulated by TGF-β and can modulate expression of multiple downstream proteins including α-smooth muscle actin (α-SMA) and collagens, which are all involved in fibrosis [5, 6]. Though heavily studied, the profibrotic effect of periostin in DCM is still far from being fully elucidated.

It is generally accepted that oxidative stress is an essential hallmark of diabetes, moreover, activation of ERK/TGF-β pathway and upregulated collagen production contributed to fibrosis [7]. Considering the relationship between TGF-β and periostin, we postulate that activation of ERK/TGF-β/periostin pathway by oxidative stress is, in part, a key event in the development of myocardial fibrosis in DCM.

Given the critical role of oxidative stress in diabetes, there are a few studies looking specially at the effect of antioxidants on DCM [8]. Resveratrol, a polyphenol present in grapes and red wine, has antioxidative effects on cardiovascular diseases [9]. Fragmentary data on animal experiments has indicated that resveratrol could prevent fibrosis in DCM via inhibition of oxidative stress [10], yet, the precise regulatory mechanisms are currently unknown.

Thus, this study was designed to test whether activation of TGF-β/periostin pathway is associated with myocardial fibrosis of DCM in STZ-induced diabetic mice and to evaluate whether resveratrol ameliorates fibrogenesis by modulating ROS/ERK/TGF-β/periostin pathway in vivo and in vitro.

## Methods

### Animals and procedures

The experimental and feeding protocols were in accordance with National Health guidelines and were approved by the Animal Care and Use Committee of the Affiliated Drum Tower Hospital of Nanjing University Medical School. C57BL/6 mice (male) were purchased from Model Animal Research Center of Nanjing University. Mice were randomly assigned into four groups containing eight mice each. Diabetes mellitus was induced by consecutive intraperitoneal administration of STZ (40 mg/kg/day, Sigma) dissolved in 0.1 M sodium citrate buffer, PH 4.5, for 5 days as described [11]. Mice with a blood glucose level above 13.9 mM at 3 days after the last STZ injection were considered as diabetic animals. One month after induction of diabetes, mice were treated with resveratrol (5 or 25 mg/kg/day i.g, Sigma) for another 2 months. Saline containing 0.5 % CMC (vehicle) was given as a control. Changes in body weight and blood glucose were recorded every 2 weeks until the animals were sacrificed.

### Masson’s trichrome staining

After fixation with 4 % paraformaldehyde in phosphate-buffered saline for 24 h, the cardiac tissues were subjected to alcoholic dehydration and embedded in paraffin. 4 μm serial sections were sliced and subjected to Masson’s trichrome staining to observe the degree of myocardial fibrosis. Collagen volume fraction (CVF) was determined by Image Pro Plus software, and the mean values of CVF were obtained by one investigator blinded to the groups.

### DHE staining

In this study, superoxide expression was detected by DHE staining. The heart sections were incubated with 2 μm/ml DHE dye (Beyotime Technology) for 30 min at 37 °C protected from light. Oxidative stress was examined and captured on immunofluorescence microscope. Red staining indicating oxidative stress was quantified using Image Pro Plus software.

### Cell culture

Primary mouse cardiac fibroblasts (mCFs) were isolated and cultured as previously described [12]. Briefly, after being excised from mice, the hearts were washed in cold PBS. The ventricles were minced and digested in Dulbecco’s modified Eagle’s medium (DMEM) containing 0.1 % collagenase type 2 (Worthington) at 37 °C with continous shaking for 30 min. After being pre-plated for 1 h at constant incubator, the unattached cells were removed and cultured in DMEM containing 10 % foetal bovine serum (FBS).

### Western blot analysis

Cells or mice heart tissues were lysed using RIPA buffer containing a 1:100 dilution of protease inhibitor and phosphatase inhibitor (Sigma). Protein concentrations were determined by a BCA protein assay (Pierce), and equal protein samples were separated by SDS-PAGE. Proteins were transferred electrophoretically to polyvinylidene difluoride membranes (Millipore), and then incubated in Tris-buffered saline containing 0.1 % Tween 20 (TBST) with 5 % milk for 1 h at room temperature. Membranes were then incubated with primary antibodies as follows: anti-p66shc (1:1000, Santa Cruz), anti-p47phox (1:1000, Santa Cruz), anti-gp91phox (1:1000, Santa Cruz), anti-α-SMA (1:1000, Abcam), anti-ERK (1:1000, Santa Cruz), anti-pERK (1:1000, Santa Cruz), anti-collagen I (1:500, Bioworld), anti-periostin (1:500, Santa Cruz) and anti-TGF-β (1:500, Santa Cruz). Anti-β-actin antibody (1:2000, Santa Cruz) was used as the internal control. After four washes in TBST, the blots were incubated with horseradish peroxidase-conjugated secondary antibodies. The washes were repeated, and the membranes were then treated with Super Signal Substrate Western Blotting Reagent (Millipore). The bands were quantified using BioRad Quantity One imaging software.

### Immunofluorescence staining

Measurement of α-SMA using immunofluorescence staining was performed as previously described [12]. Briefly, after being placed in 24 well plates, mCFs were washed with PBS twice and then were blocked with 1 % bovine serum albumin at room temperature for 30 min. Then the cells were incubated overnight with rabbit anti-α-SMA primary antibody (1:100, Abcam). After being washed with PBS, the cells were performed with goat anti-rabbit secondary antibody conjugated to Alexa Fluor 488 (1:400, Invitrogen) for 1 h under dark condition. Finally, the cells were counterstained with DAPI (Sigma) for 10 min, and were analyzed with an immunofluorescence microscope.

### Measurement of intracellular ROS in fibroblasts

Intracellular ROS generation was measured using fluorescent probe 2′,7′-dichlorofluorescin diacetate (DCFH-DA) as previously described [12]. Briefly, mCFs were plated in 24 well plates at a density of 5 × 10^5^ cells /well. After different treatments, medium was removed and the cells were washed with PBS. A solution of 5 μM DCFH-DA probe (Sigma) in serum free medium was then added for 30 min at 37 °C. Then intracellular ROS were detected by immunofluorescence microscope.

### MTT assay

mCFs were seeded in 96-well culture plates at 10^5^ cells/well. After serum starvation, the cells were treated as indicated in the figure legends. The cells were washed twice with PBS, and were resuspended in 1 ml DMEM without FBS. After incubation with 5 mg/ml MTT reagent (Beyotime Technology), the cells were solubilized in DMSO to solubilize the formazan crystals. The absorption was measured by spectrophotometry at 490 nm.

### Statistical analysis

Difference between two groups was analyzed by student’s t test (normally distributed) or Mann–Whitney (non-normally distributed). One-way analysis of variance (normally distributed) or Kruskal-Wallis test (non-normally distributed) was used between more than two groups. Values of *p* < 0.05 were considered significant. Analyses were performed with SPSS 21.0.

## Results

### Changes of body weight and blood glucose with resveratrol treatment

**Fig 1:**
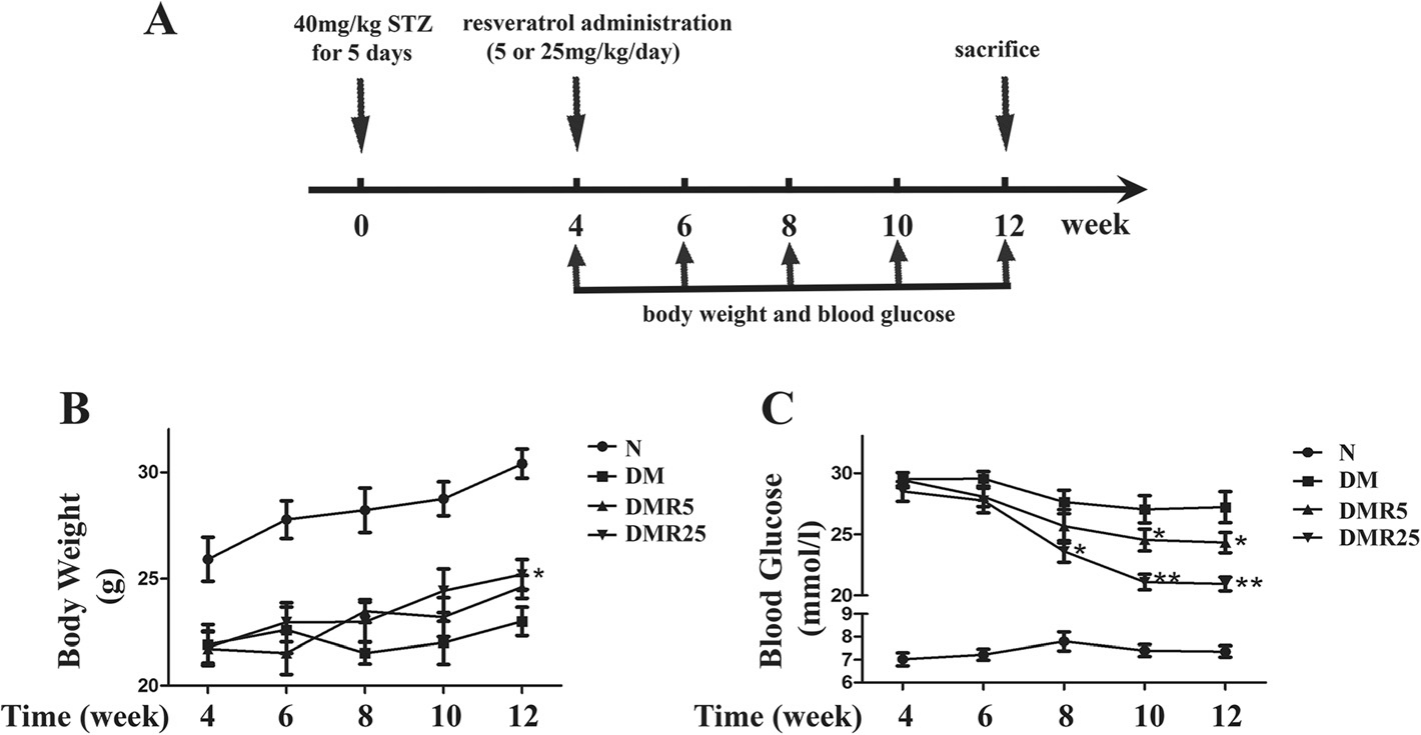
Body weight and blood glucose levels in different groups. **a** Protocol for the induction of diabetes and the treatment of resveratrol. **b** Body weight in non-diabetic, diabetic and resveratrol-treated diabetic mice. **c** Blood glucose in non-diabetic, diabetic and resveratrol-treated diabetic mice. *n* = 8 in each group; *p < 0.05 vs DM group; **p < 0.01 vs DM group

Four weeks after induction of diabetes, the diabetic mice administrated with solvent or resveratrol (5 mg/kg/day or 25 mg/kg/day) were divided into DM group, DMR5 group and DMR25 group respectively (Fig. 1a). The mice without injection of STZ were used as control (N group). Changes of body weight and blood glucose were shown in Fig. 1b, Compared with DM group, body weight of N group increased with time passing by, while resveratrol treatment produced a small but significant increase in body weight in DMR25 group at the 12th week (25.20 ± 1.57 vs 23.00 ± 1.48, *p* < 0.05). Blood glucose was significantly higher in diabetic mice compared with non-diabetic mice. However, resveratrol-treated mice showed a sustained decrease in blood glucose from 8th week to the end of the study period (Fig. 1c).

### Resveratrol attenuated myocardial fibrosis and oxidative stress in STZ-induced diabetic heart

**Fig 2:**
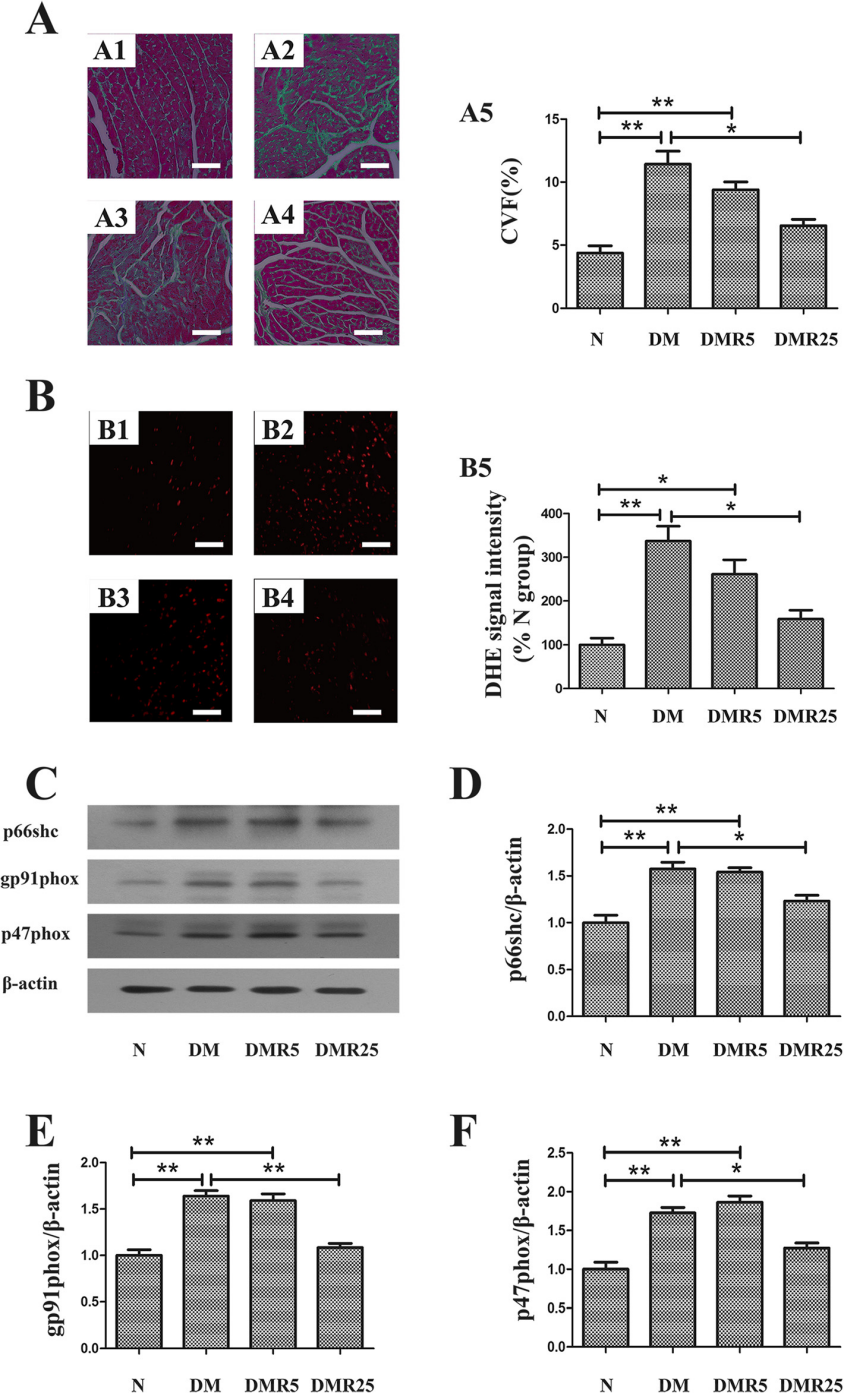
Effect of resveratrol on myofibrosis and oxidative stress in diabetic hearts. **a** Representative microphotographs of Masson’s trichrome staining heart section of four groups (A1, N group; A2, DM group; A3, DMR5 group; A4, DMR25 group; A5, Quantitative analysis of CVF). **b** Representative microphotographs of DHE staining in heart sections in each group (B1, N group; B2, DM group; B3, DMR5 group; B4, DMR25 group; B5, Quantitative analysis of DHE staining). **c** Western blot of oxidative stress related proteins in different groups. **d** Quantitative analysis of p66shc. **e** Quantitative analysis of gp91phox. **f** Quantitative analysis of p47phox. *n* = 8 in each group; *p < 0.05 between two groups; **p < 0.01 between two groups; bar = 50 μm

To assess the impact of resveratrol on diabetes-induced myocardial fibrosis, Masson’s trichrome staining was performed. As shown in Fig. 2a, myocardial fibrosis was significantly increased in the diabetic mice compared with control mice (11.42 ± 1.79 vs 4.37 ± 0.99, *p* < 0.01). Interestingly, high dose (25 mg/kg/day) resveratrol treatment remarkably suppressed the diabetes-induced fibrosis (6.54 ± 0.87 vs 11.42 ± 1.79, *p* < 0.05), however, low dose (5 mg/kg/day) resveratrol showed no effect on diabetes-induced myocardial fibrosis(9.39 ± 1.08 vs 11.42 ± 1.79, p > 0.05). To evaluate the effects of resveratrol on oxidative stress in diabetic hearts, superoxide expression was detected by DHE staining. Fig 2b depicted significantly higher DHE expression in hearts of DM group, which was significantly inhibited in DMR25 group (159.10 ± 34.60 vs 337.00 ± 59.20, *p* < 0.05). Furthermore, the antioxidative effect of resveratrol was supported by western blot assay, in which several oxidative stress related proteins, such as p47phox, gp91phox and p66shc were detected. Diabetic mice exhibited increased expressions of these proteins compared with control mice, while treatment with resveratrol partly normalized their expressions in diabetic mice (Fig. 2c, d, e, f).

### Resveratrol regulated ERK/TGF-β/periostin pathway in diabetic hearts

**Fig 3:**
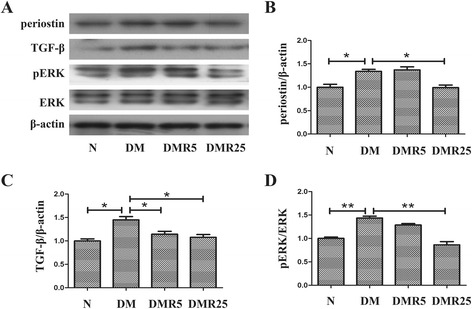
Effect of resveratrol on ERK/TGF-β/periostin signaling pathway in hearts of STZ-induced diabetic mice. **a** Representative immunoblots of pERK, ERK, TGF-β and periostin in the hearts of each group. **b** Quantitative analysis of periostin protein expression. **c** Quantitative analysis of TGF-β protein expression. **d** Quantitative analysis of ERK activity. *n* = 8 in each group; *p < 0.05 between two groups; **p < 0.01 between two groups

To study how resveratrol modulates myocardial fibrosis induced by diabetes, western blot was performed to analyse the protein expressions of pERK, ERK, TGF-β and periostin, which were considered to be involved in fibrosis. As shown in Fig. 3, expressions of TGF-β and periostin were significantly elevated in diabetic mice compared with control mice (1.45 ± 0.13 vs 1.00 ± 0.07, *p* < 0.05 and 1.34 ± 0.07 vs 1.00 ± 0.11, *p* < 0.05 respectively), and the upregulation of periostin was attenuated by treatment of high dose resveratrol (Fig. 3b; 0.99 ± 0.09 vs 1.34 ± 0.07, *p* < 0.05), while TGF-β was inhibited by both low and high dose resveratrol (Fig. 3c; 1.14 ± 0.11 vs 1.45 ± 0.13, *p* < 0.05 and 1.08 ± 0.10 vs 1.45 ± 0.13, *p* < 0.05 respectively). As well as periostin, activation of ERK was also reduced in DMR25 group compared with DM group (Fig. 3d; 0.86 ± 0.12 vs 1.43 ± 0.07, *p* < 0.05).

### Resveratrol inhibited high glucose (HG)-induced differentiation of mCFs via diminution of ROS production

It is widespread agreement that activity or differentiation of fibroblasts is a critical feature of progressive fibrosis and is associated with oxidative stress, therefore, it is rational to presume that resveratrol may suppress fibroblasts activity via inhibition of oxidative stress. So an in vitro assay was performed to ascertain the effect of resveratrol on differentiation of mCFs and to explore the underlying mechanism.

**Fig 4:**
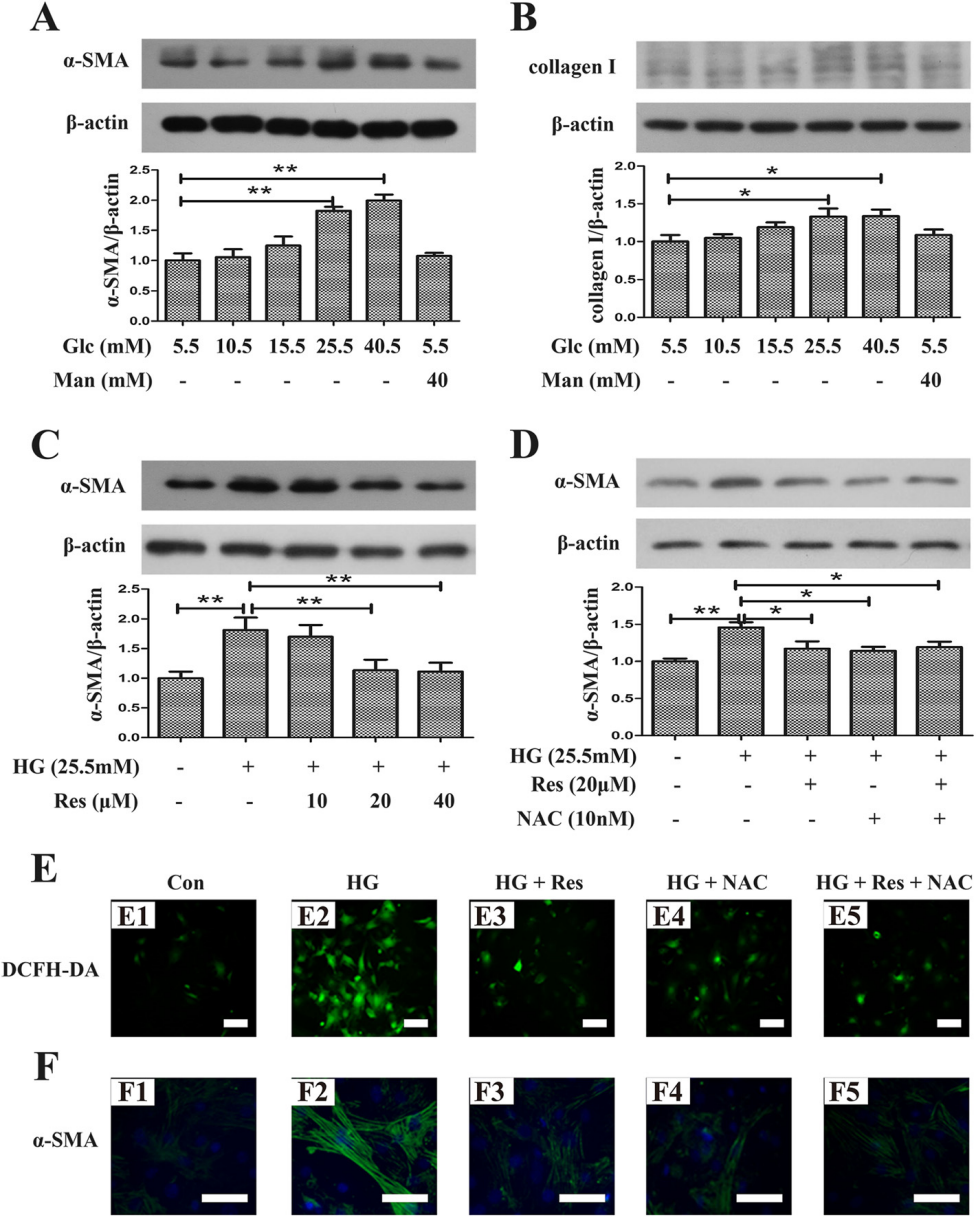
HG-induced differentiation of mCFs was attenuated by resveratrol and NAC. **a** Effect of different glucose concentrations on the expression of α-SMA in mCFs. **b** Effect of different glucose concentrations on the expression of collagen I in mCFs. **c** Resveratrol attenuated upregulated α-SMA expression induced by HG in a dose dependent manner. **d** NAC mimicked the effect of resveratrol on expression of α-SMA induced by HG. **e** Immunofluorescence assay showed inhibition of ROS by resveratrol and NAC (E1, control group; E2, HG group; E3, HG + Res group; E4, HG + NAC group; E5, HG + Res + NAC group). **f** Upregulated α-SMA expression induced by HG was attenuated by resveratrol and NAC using immunofluorescence assay(F1, control group; F2, HG group; F3, HG + Res group; F4, HG + NAC group; F5, HG + Res + NAC group). * *p* < 0.05 between two groups; **p < 0.01 between two groups; bar = 50 μm

To determine the effect of HG on differentiation of fibroblasts in vitro, mCFs were incubated with 5.5–45.5 mmol/l glucose for 24 h, and mannitol was used as a control to exclude the effect of hyperosmosis. Western blot analysis revealed an upregulation of both α-SMA and collagen I in glucose-induced mCFs in a dose dependent manner, and it reached a significant difference at 25.5 mmol/l (Fig. 4a, b; 2.00 ± 0.16 vs 1.00 ± 0.20, *p* < 0.01 and 1.34 ± 0.15 vs 1.00 ± 0.15, *p* < 0.05 respectively). Thus, mCFs stimulated with 25.5 mmol/l glucose for 24 h were selected for the following studies. Then, HG-stimulated mCFs were pretreated with resveratrol at different concentrations (10, 20, 40 μmol/l). Resveratrol significantly normalized the elevated expression of α-SMA induced by HG at 20 μmol/l (Fig. 4c; 1.14 ± 0.18 vs 1.81 ± 0.21, *p* < 0.01), which was similar to treatment of NAC (Fig. 4d). Therefore, immunofluorescence assay was performed by incubation of resveratrol at 20 μmol/l and NAC in the next study. As illustrated in Fig. 4e and f, addition of NAC blunted ROS generation using DCFH-DA, which was paralleled by the diminution of α-SMA level using immunofluorescence assay, and the effects were mimicked by the addition of resveratrol. Additionally, the effects of combined incubation with resveratrol and NAC on HG-induced mCFs were similar to that with either agent alone.

### Resveratrol inhibited HG induced mouse fibroblasts proliferation via inhibition ofROS/ERK pathway

Proliferation of fibroblasts has been recognized as an important component in the progress of fibrosis, thus, the effect of resveratrol on proliferation of mCF induced by HG was investigated in this study.

**Fig 5:**
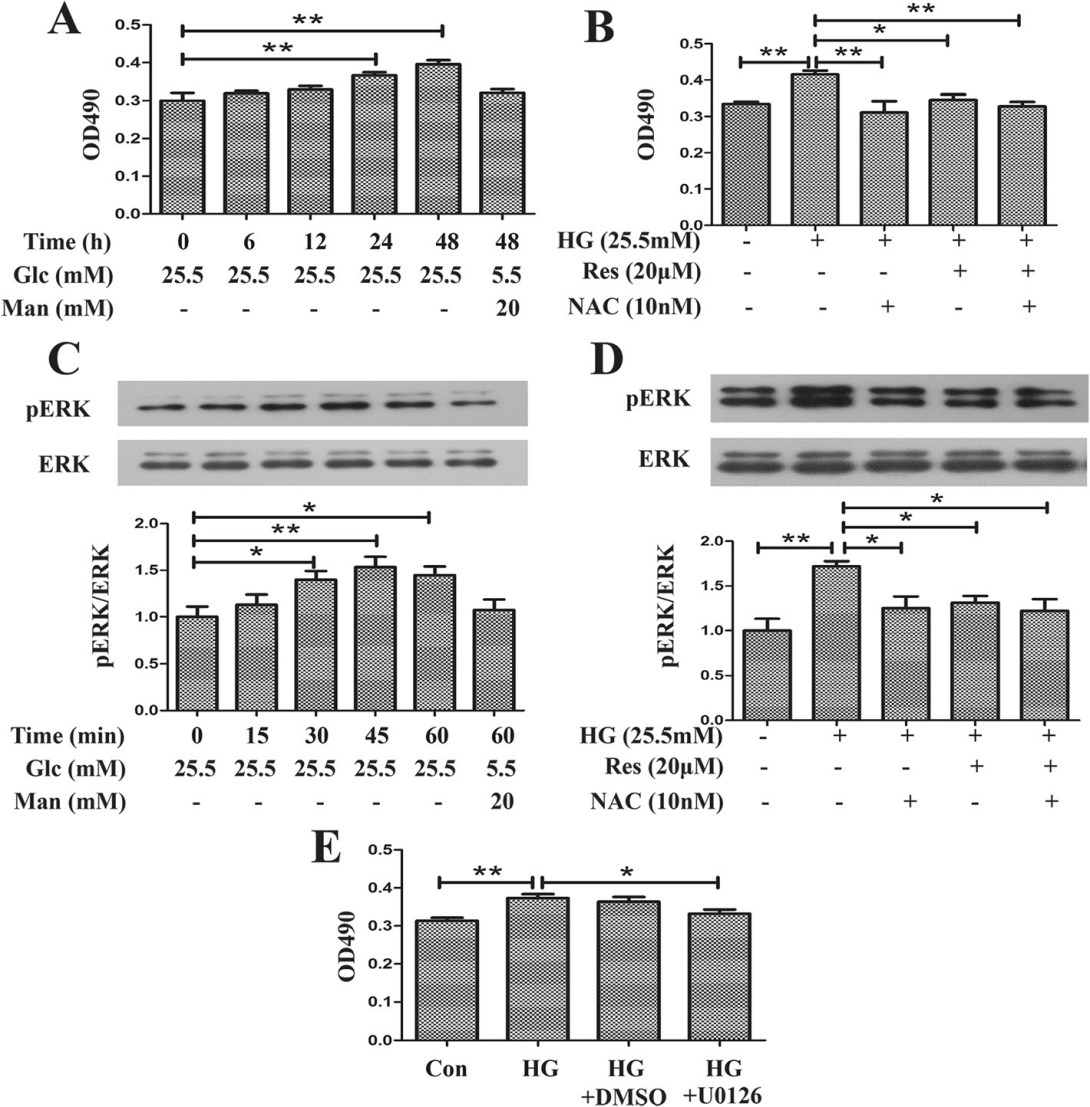
Resveratrol retarded HG-induced proliferation of mCFs via inhibition of ROS/ERK pathway. **a** HG time dependently promoted proliferation of mCFs. **b** Resveratrol as well as NAC inhibited proliferation of HG-induced mCFs. **c** HG upregulated ERK activity in a time dependent manner. **d** Resveratrol and NAC suppressed the elevated ERK activity induced by HG. **e** HG-induced proliferation of mCFs was normalized by U0126. *p < 0.05 between two groups; **p < 0.01 between two groups; bar = 50 μm

The results of MTT assay showed that there was a slight but significant increase of mCFs induced by HG for 24 h (Fig. 5a). So we took 24 h as a proper time to explore the effect of resveratrol on proliferation of mCFs. As shown in Fig. 5b, the OD490 values were reversed to normal by the the administration of resveratrol, NAC, or both of them.

It is widely assumed that ERK has a positive regulatory role in proliferation response to an array of stimuli, so western blot was performed to study the role of ERK in amelioration of proliferation by resveratrol. As illustrated in Fig. 5c, HG (25.5 mmol/l) induced significant increase of about 50 % in phosphorylation of ERK, while a non-significant increase was noted for equal osmolality (glucose (Glc) 5.5 mmol/l + mannitol (Man) 20 mmol/l). HG-induced activity of ERK was abrogated at least partly by pretreatment of resveratrol or NAC (1.17 ± 0.17 vs 1.46 ± 0.12, *p* < 0.05 and 1.14 ± 0.09 vs 1.46 ± 0.12, *p* < 0.05), while compounds of resveratrol and NAC did not exert a synergistic effect on expression of pERK (Fig. 5d). In addition, HG-induced proliferation of mCFs was suppressed by pretreatment of U0126 (10 μmol/l), an inhibitor of ERK-activating kinases (MEK) (0.33 ± 0.02 vs 0.37 ± 0.01, *p* < 0.05) (Fig. 5e).

### ERK/TGF-β/periostin pathway is involved in differentiation of mCFs

**Fig 6:**
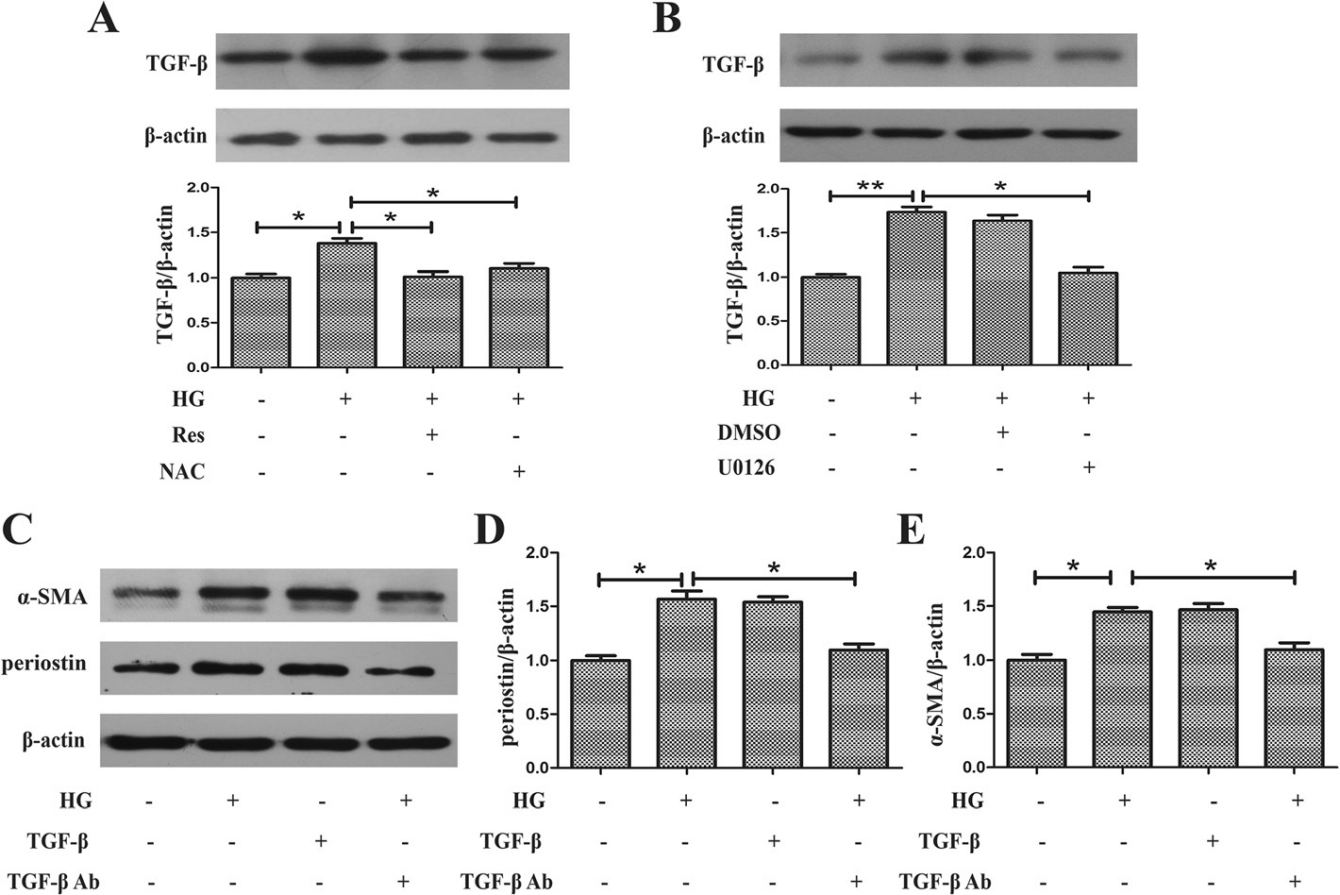
HG increased expression of periostin via ROS/ERK/TGF-β pathway. **a** Resveratrol inhibited expression of TGF-β in HG-treated myofibroblasts as well as NAC. **b** Treatment with U0126 suppressed the elevated TGF-β induced by HG in myofibroblasts. **c** HG-induced increased expressions of periostin and α-SMA were suppressed by pretreatment with TGF-β neutralizing antibody. **d** Quantitative analysis of periostin. **e** Quantitative analysis of α-SMA. *p < 0.05 between two groups; **p < 0.01 between two groups

To investigate the role of ERK/TGF-β pathway in ROS-induced periostin expression in more detail, we performed an in vitro experiment on mCFs using neutralizing antibody. Fig 6a showed that resveratrol and NAC had a significant inhibitory effect on elevated expression of TGF-β induced by HG (1.01 ± 0.10 vs 1.38 ± 0.10, *p* < 0.05 and 1.10 ± 0.10 vs 1.38 ± 0.10, *p* < 0.05 respectively). Then we asked whether HG regulated expression of TGF-β via ERK mediated pathway. It was observed that HG-induced upregulation of TGF-β was suppressed by U0126, an inhibitor of MEK (1.05 ± 0.11 vs 1.74 ± 0.10, *p* < 0.05), suggesting the involvement of ERK in HG-induced TGF-β expression (Fig. 6b). As shown in Fig. 6c, d, e, after being cultured with TGF-β, mCFs exhibited elevated α-SMA and periostin expressions (1.10 ± 0.11 vs 1.45 ± 0.07, *p* < 0.05 and 1.10 ± 0.10 vs 1.57 ± 0.13, *p* < 0.05 respectively), which were similar to the effects of HG. Furthermore, pretreatment with TGF-β neutralizing antibody partly corrected the dysregulations of α-SMA and periostin induced by HG (Fig. 6d, e; 1.10 ± 0.11 vs 1.47 ± 0.10, *p* < 0.05 and 1.10 ± 0.10 vs 1.54 ± 0.08, *p* < 0.05 respectively).

## Discussion

Here we show that periostin is involved in myocardial fibrosis in STZ-induced type 1 diabetic mice. Furthermore, resveratrol suppresses HG-induced differentiation of mCFs via ROS/ERK/TGF-β/periostin pathway and suppresses HG-induced proliferation of mCFs via ROS/ERK pathway. Our results suggest that ROS/ERK/TGF-β/periostin pathway may contribute to myocardial fibrosis in DCM and resveratrol has a therapeutic potential in ameliorating these processes.

Resveratrol is a naturally existing phytoalexin found largely in grapes. It exerts potent antioxidant effects and cardioprotective effects, including myocardial ischemia-reperfusion injury [13], cardiomyocyte hypertrophy [10], myocardial inflammation [14] and atherosclerosis [9]. Here, our in vitro experiments demonstrated that resveratrol treated group manifested less fibrosis level than no resveratrol treated group, evidenced by a significant decrease in CVF using Masson’s trichrome staining. This was accompanied by decreased oxidative stress evidenced by downregulation of p47phox, p66shc, gp91phox and ROS level. Supporting these statements, Qin et al. demonstrated that reduction of oxidative stress was a potential mechanism contributed to the beneficial effects of resveratrol in diet-induced metabolic heart disease in mice [10]. However, the exact mechanisms remain poorly understood, which was heavily studied in this present study.

Periostin, a secreted extracellular matrix (ECM) protein, is upregulated dramatically under transverse aortic constriction (TAC) stress [2, 15, 16], myocardial infarction [3, 17] or heart failure [1]. An accumulating body of evidence suggests that periostin plays an important role in fibrosis by regulating ECM molecules such as collagen and fibronectin [18]. The importance of periostin in fibrogenesis was also documented in studies utilizing periostin knock-out animal, where deletion of periostin dramatically suppressed muscular fibrosis [19]. However, to our knowledge, there was little information about its profibrotic effect in DCM to date. In our diabetic mice, periostin expression was significantly increased in diabetic hearts compared with nondiabetic hearts. In cultured mCFs, HG induced the protein level of periostin, which was accordance to previous studies of rat cardiac fibroblasts [20]. Interestingly, administration of resveratrol partly blunted these changes, suggesting that resveratrol may suppress interstitial fibrosis via inhibition of periostin.

CFs, the predominant cells in ventricular, mediates cardiac fibrosis via their proliferation and differentiation into myofibroblasts. Proliferation and differentiation of mCFs to myofibroblast phenotype can contribute to excessive secretion of ECM proteins and then promote cardiac fibrosis [21]. So the precise mechanisms how resveratrol suppresses the proliferation and differentiation of mCFs induced by HG were also investigated in this study.

Evidence has accumulated that ERK signaling activation was involved in fibroblast proliferation under various stimulation [22–24]. Here, we found a significantly increased proliferation and activity of ERK under high glucose condition, while U0126, an inhibitor of MEK, ameliorated mCFs proliferation induced by HG. These results suggested that HG promoted mCFs proliferation via ERK related signaling pathway, which supported previous observations that ERK was an important mediator in fibroblast proliferation induced by HG, angiotensin II (AngII), TGF-β or basic fibroblast growth factor (bFGF) [22–24]. However, the role of ERK in the amelioration of HG-induced mCFs proliferation by resveratrol is poorly understood. As mentioned above, oxidative stress plays a central role in fibrosis of DCM. Therefore, we postulated that ROS production was an important contributing factor for active ERK in DCM. In this investigation, we reported that administration of resveratrol significantly decreased ERK phosphorylation both in vitro and in vivo. Furthermore, NAC, an antioxidant, mimicked the effect of resveratrol. Collectively, these data demonstrated that resveratrol mitigated HG-induced mCFs proliferation via inhibition of ROS/ERK pathway. In favor of these results, Li et al. recently found that α-lipoic acid (ALA), another antioxidant, favorably shifted redox homeostasis and suppressed ERK activation in diabetic hearts [8].

Differentiation of myofibroblast, as evidenced by α-SMA and collagens expression, is largely mediated by TGF-β [21]. TGF-β has been proposed a profibrotic factor to promote the synthesis of ECM and contribute to cardiac fibrosis [21, 25, 26]. In addition, it is generally accepted that AngII induced increased expression of TGF-β in CFs [27, 28], and several studies have shed light on the effect of ERK signaling on AngII-induced TGF-β expression [28–30]. Likewise, in this study, addition of MEK inhibitor U0126 normalized the elevated TGF-β induced by HG, suggesting that HG increased expression of TGF-β via ERK signaling pathway. Furthermore, in vitro and in vivo experiments demonstrated that resveratrol treatment significant abolished the upregulation of pERK and TGF-β in diabetic heart or HG condition, which was in line with two novel studies showing that resveratrol inhibited high glucose-induced TGF-β in cardiac fibroblasts [25, 26]. These results suggested that resveratrol could ameliorate TGF-β expression via ROS/ERK pathway.

In addition to Smad2/3 activation, increasing evidence supported the involvement of periostin in TGF-β induced fibrosis [6, 28, 31–33]. Most studies agreed that periostin was a downstream signal molecule of TGF-β and participated in TGF-β-induced cardiac fibrosis [5, 34]. In this present study, anti-TGF-β antibody attenuated HG-induced periostin expression, which showed the involvement of TGF-β in upregulated periostin expression induced by HG. To our knowledge, this study is the first attempt to block the upregulated expression of periostin as well as myocardial fibrosis in vivo by daily treatment with resveratrol. Thus it provides a novel mechanism by which resveratrol inhibited diabetes-induced fibrosis in myocardium.

## Conclusions

Taken all the results above together, our investigation demonstrates that periostin is a central element in diabetes related myocardium fibrosis, which is prevented by resveratrol via inhibition of ROS/ERK/TGF-β pathway.

## Abbreviations

DCM: Diabetic cardiomyopathy
STZ: Streptozocin
CMC: Carboxymethyl cellulose
DHE: Dihydroethidium
MTT: 3-(4,5)-dimethylthiahiazo (−z-y1)-3,5-di-phenytetrazoliumromide
ROS: Reactive oxygen species
ERK: Extracellular regulated kinase
TGF-β: Transforming growth factor β
DM: Diabetes mellitus
α-SMA: α-smooth muscle actin
CVF: Collagen volume fraction
mCFs: Mouse cardiac fibroblasts
DMEM: Dulbecco’s modified Eagle’s medium
FBS: Foetal bovine serum
TBST: Tris-buffered saline containing 0.1 % Tween 20
DCFH-DA: 2′,7′-dichlorofluorescin diacetate
HG: High glucose
Glc: Glucose
Man: Mannitol
ECM: Extracelluar matrix
TAC: Transverse aortic constriction
AngII: Angiotensin II
bFGF: Basic fibroblast growth factor
ALA: α-lipoic acid
